# Comparison of Storage Conditions for Human Vaginal Microbiome Studies

**DOI:** 10.1371/journal.pone.0036934

**Published:** 2012-05-24

**Authors:** Guoyun Bai, Pawel Gajer, Melissa Nandy, Bing Ma, Hongqiu Yang, Joyce Sakamoto, May H. Blanchard, Jacques Ravel, Rebecca M. Brotman

**Affiliations:** 1 Institute for Genome Sciences, University of Maryland School of Medicine, Baltimore, Maryland, United States of America; 2 Department of Microbiology and Immunology, University of Maryland School of Medicine, Baltimore, Maryland, United States of America; 3 Department of Obstetrics, Gynecology and Reproductive Sciences, University of Maryland School of Medicine, Baltimore, Maryland, United States of America; 4 Department of Epidemiology and Public Health, University of Maryland School of Medicine, Baltimore, Maryland, United States of America; Columbia University, United States of America

## Abstract

**Background:**

The effect of storage conditions on the microbiome and metabolite composition of human biological samples has not been thoroughly investigated as a potential source of bias. We evaluated the effect of two common storage conditions used in clinical trials on the bacterial and metabolite composition of the vaginal microbiota using pyrosequencing of barcoded 16S rRNA gene sequencing and ^1^H-NMR analyses.

**Methodology/Principal Findings:**

Eight women were enrolled and four mid-vaginal swabs were collected by a physician from each woman. The samples were either processed immediately, stored at −80°C for 4 weeks or at −20°C for 1 week followed by transfer to −80°C for another 4 weeks prior to analysis. Statistical methods, including Kolmogorovo-Smirnov and Wilcoxon tests, were performed to evaluate the differences in vaginal bacterial community composition and metabolites between samples stored under different conditions. The results showed that there were no significant differences between samples processed immediately after collection or stored for varying durations. ^1^H-NMR analysis of the small molecule metabolites in vaginal secretions indicated that high levels of lactic acid were associated with *Lactobacillus*-dominated communities. Relative abundance of lactic acid did not appear to correlate with relative abundance of individual *Lactobacillus* sp. in this limited sample, although lower levels of lactic acid were observed when *L. gasseri* was dominant, indicating differences in metabolic output of seemingly similar communities.

**Conclusions/Significance:**

These findings benefit large-scale, field-based microbiome and metabolomic studies of the vaginal microbiota.

## Introduction

The ability to process human biological specimens immediately after collection is not feasible in large field-based epidemiologic studies, and therefore the effect on storing samples for extended periods of time is always in question. Very little information is available on the effect of storage conditions on the microbes associated with these samples. Any effect on their representiveness could potentially affect studies of the human microbiome. Prior studies on soil, fecal and urine samples have shown conflicting results of the effect of storage condition on bacterial composition [1], [2], [3], [4], [5], [6], [7] and the metabolome [8], [9], [10], [11], [12], [13], [14]. The effect of storage condition appears to depend on the sample type, duration of storage and the analytical method used. No such study has been performed on vaginal specimens.

In the present study, clinician-collected vaginal specimens were obtained to investigate the effect of two commonly used storage conditions on the bacterial and metabolite composition of the vaginal microbiota. We used culture-independent pyrosequencing of barcoded 16S rRNA gene sequencing analysis to establish the bacterial composition and ^1^H NMR spectroscopy to characterize the vaginal metabolome. Analysis of the 16S rRNA gene is the current standard method to study the composition of the human microbiome[15]. ^1^H NMR spectroscopy allows for the simultaneous detection of 30–50 small molecule metabolites, requires little preparation and exhibits excellent precision and reproducibility [16], [17].

## Materials and Methods

Eight women were recruited from the Maryland Women's Health Obstetrics and Gynecology practice at the University of Maryland School of Medicine in June 2010. Inclusion criteria were adult women over age 18 who were not menstruating and were not pregnant. Using validated protocols [18], [19], a gynecologist collected four mid-vaginal swabs during a routine speculum exam. The study was approved by Institutional Review Boards at the University of Maryland School of Medicine. All participants provided written informed consent.

**Table 1:** Characteristics of women recruited for the comparison of storage conditions for vaginal secretion samples, Baltimore, MD (n = 8).

	N	%
Age*	40.5 (range: 25-63)	
Ethnicity		
Black	5	62.5
White	3	37.5
Use of feminine hygiene products in prior 2 months	6	75
pH>4.5	6	75
Marital status		
divorced	3	37.5
married	1	12.5
never married	4	50
Lifetime number of sex partners*	8 (range: 3–60)	

* Median, range in parantheses.

**Figure 1:**
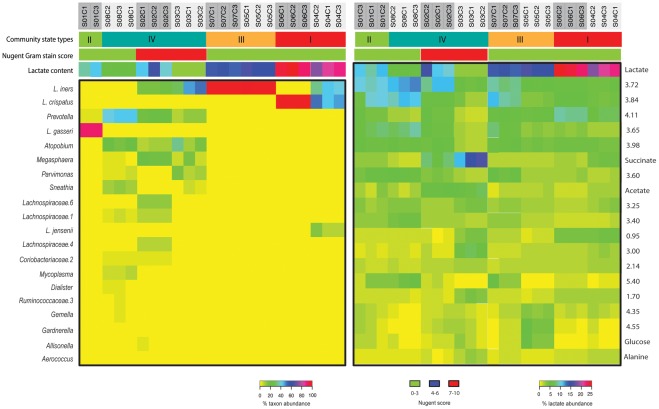
Heatmap of the relative abundance of microbial taxa characterized by 16S rRNA gene sequencing (left) and metabolic content featured by NMR (right) in samples from 8 participants (S01-S08) stored under three different conditions (C1-C3). The community state types, the relative content of lactic acid and Nugent score are also shown. The unassigned NMR integration regions were labeled by chemical shift.

To characterize the metabolic composition, three dry dacron swabs (Starplex Scientific Starswab II Collection and Transport Systems) were collected and stored dry in a tube. For characterization of the vaginal bacterial composition, one ESwab (Copan Liquid Amies Elution Swab Collection and Transport System) was collected and then used to create a vaginal smear followed by storage in modified Liquid Amies solution. The vaginal smears were heat-fixed and Gram-stained, then blinded and evaluated in random order by microcroscopy. A score of 0–10 was assigned by an experienced microbiologist using the standardized method described by Nugent *et* *al*.[20] Nugent's scores are composite scores based on the cellular morphologies of the bacteria present in a sample. A score of 0–3 was designated normal, 4–6 as intermediate and 7–10 as a high score indicative of bacterial vaginosis (BV).

The swabs were immediately stored on ice and transported to the laboratory for processing and cold-storage. Each ESwab was placed in 1 ml Liquid Amies solution in the clinic. The Liquid Amies solution was then divided in three sub-aliquots within five minutes prior to sample processing. Three cold chain protocols were tested on 1 dry dacron swab and 1 liquid amies sub-aliquot: 1). Samples processed directly within three hours of collection represented Sample Storage Condition #1(C1); 2) samples stored for 4 weeks at −80°C for 4 weeks before being processed represented Sample Storage Condition #2(C2); and 3) samples stored for 1 week at −20°C for 1 week, then transferred to −80°C for an additional 4 weeks before processing represented Sample Storage Condition #3(C3). Condition #3 mimics the storage condition often used in field-based studies in which subjects self-collect swabs at home and store them at −20°C before transporting the swabs to the clinic for storage at −80°C prior to processing. The −20°C freezer was a frost-free freezer to better simulate household storage in a clinical trial.

**Table 2:** Microbial community composition as a percentage of the top 20 most abundant taxa present in three samples stored under different conditions (C1, C2 and C3) collected from each of 8 subjects (S01-S08).

Subject ID	Condition	Community state type	Total no. of reads	Percentage (%) of taxa																			
				*L. iners*	*L. crispatus*	*Prevotella*	*L. gasseri*	*Atopobium*	*Megasphaera*	*Parvimonas*	*Sneathia*	*Lachnospiraceae.6*	*Lachnospiraceae.1*	*L. jensenii*	*Lachnospiraceae.4*	*Coriobacteriaceae.2*	*Mycoplasma*	*Dialister*	*Ruminococcaceae.3*	*Gemella*	*Gardnerella*	*Allisonella*	*Aerococcus*
S01	C1	II	5788	0.3	0.0	3.0	90.7	0.1	0.0	0.0	0.0	0.0	0.0	0.0	0.0	0.0	0.0	0.0	0.0	0.0	0.0	0.0	0.0
	C3	II	8473	1.0	0.0	2.8	89.5	0.1	0.0	0.0	0.0	0.0	0.0	0.1	0.0	0.0	0.0	0.0	0.0	0.0	0.0	0.0	0.0
S02	C1	IV	5784	14.9	0.0	17.4	0.0	6.7	18.9	0.5	0.3	11.5	6.8	0.0	7.7	3.3	0.1	0.8	0.7	0.1	0.6	2.3	0.9
	C2	IV	9059	14.1	0.0	18.3	0.0	9.3	17.8	0.3	0.2	11.5	7.0	0.0	7.0	3.1	0.0	0.6	0.6	0.2	1.0	1.1	0.9
	C3	IV	6610	16.5	0.0	19.4	0.0	8.0	16.1	0.4	0.5	11.6	6.7	0.0	7.0	2.2	0.0	0.6	0.6	0.2	0.9	1.1	1.0
S03	C1	IV	7302	46.0	0.0	11.0	0.0	9.5	14.6	6.6	4.9	0.2	0.2	0.0	0.0	0.3	0.9	0.8	0.4	0.5	0.1	1.0	0.4
	C2	IV	10454	50.8	0.0	10.8	0.0	15.4	4.5	8.0	2.1	0.4	0.5	0.0	0.0	0.5	0.3	0.6	0.4	0.8	0.6	0.5	0.9
	C3	IV	18192	28.6	0.0	6.8	0.0	32.5	4.2	14.6	1.5	0.4	0.5	0.0	0.0	0.7	0.4	0.5	1.0	1.6	1.1	0.4	0.9
S04	C1	I	7129	42.8	42.9	0.1	0.2	0.0	0.0	0.0	0.0	0.0	0.0	7.6	0.0	0.0	0.0	0.0	0.0	0.0	0.0	0.0	0.1
	C2	I	4336	32.8	49.6	0.1	0.7	0.0	0.0	0.0	0.0	0.0	0.0	12.5	0.0	0.0	0.0	0.0	0.0	0.0	0.0	0.0	0.2
	C3	I	8740	39.6	46.6	0.1	0.3	0.0	0.0	0.0	0.0	0.0	0.0	6.9	0.0	0.0	0.0	0.0	0.0	0.0	0.0	0.0	0.1
S05	C1	III	6371	96.4	0.0	0.0	0.0	0.0	0.0	0.0	0.0	0.0	0.0	0.6	0.0	0.0	0.0	0.0	0.0	0.0	0.0	0.0	0.0
	C2	III	7491	97.5	0.0	0.0	0.1	0.0	0.0	0.0	0.0	0.0	0.0	0.8	0.0	0.0	0.0	0.0	0.0	0.0	0.0	0.0	0.0
	C3	III	8484	97.4	0.0	0.0	0.0	0.0	0.0	0.0	0.0	0.0	0.0	0.4	0.0	0.0	0.0	0.0	0.0	0.0	0.0	0.0	0.0
S06	C1	I	7574	0.0	98.0	0.0	0.0	0.0	0.0	0.0	0.0	0.0	0.0	0.2	0.0	0.0	0.0	0.0	0.0	0.0	0.0	0.0	0.0
	C2	I	8151	0.0	98.9	0.0	0.0	0.0	0.0	0.0	0.0	0.0	0.0	0.1	0.0	0.0	0.0	0.0	0.0	0.0	0.0	0.0	0.0
	C3	I	8174	0.0	98.3	0.0	0.0	0.0	0.0	0.0	0.0	0.0	0.0	0.1	0.0	0.0	0.0	0.0	0.0	0.0	0.0	0.0	0.0
S07	C1	III	6820	97.2	0.0	0.0	0.0	0.0	0.0	0.0	0.0	0.0	0.0	0.0	0.0	0.0	0.0	0.0	0.0	0.0	0.0	0.0	0.0
	C2	III	7315	97.6	0.0	0.0	0.0	0.0	0.0	0.0	0.0	0.0	0.0	0.0	0.0	0.0	0.0	0.0	0.0	0.0	0.0	0.0	0.0
	C3	III	5445	98.4	0.0	0.0	0.0	0.0	0.0	0.0	0.0	0.0	0.0	0.0	0.0	0.0	0.0	0.0	0.0	0.0	0.0	0.0	0.0
S08	C1	IV	10328	1.7	0.0	42.2	0.0	14.9	3.4	3.7	10.2	0.1	4.3	0.0	0.1	2.8	4.0	2.0	2.6	2.0	1.2	0.1	0.5
	C2	IV	4321	1.3	0.0	41.3	0.0	17.9	2.5	3.1	8.8	0.2	3.0	0.0	0.1	3.0	4.6	2.1	1.2	1.6	0.9	0.3	0.5
	C3	IV	4342	1.5	0.0	42.3	0.0	18.7	1.8	4.5	9.1	0.3	3.2	0.0	0.1	3.0	6.7	1.5	1.5	1.3	0.7	0.2	0.2

At the end of each storage period, whole genomic DNA was extracted from the Liquid Amies solution aliquots using the Zymo fecal DNA extraction kit with modifications (details below). ^1^H NMR metabolomic analyses were performed using the dacron Starplex swab samples. There were 23 samples available for bacterial composition analysis and 24 samples for NMR analysis. One sample failed to produce a 16S rRNA gene amplicon due to low DNA concentration. All subject ID#s were double blinded for publication.

### Composition of vaginal bacterial communities

#### (i) DNA extraction and purification

Fresh samples were kept chilled on ice until processed. Frozen samples were thawed on ice and kept chilled until processed. A 0.3 ml Amies aliquot was transferred to a 1.5 ml tube containing 0.1 mm silica beads (FastPrep Lysing Matrix B tube (Bio101)) and stored on ice before whole-genomic DNA extraction. Briefly, 650 µl of 1×phosphate buffered saline (PBS) containing 50 µl lyzosyme (10 mg/ml), 6 µl of mutanolysin (25,000 U/ml; Sigma-Aldrich) and 3 µl of lysostaphin (4,00 U/ml in sodium acetate; Sigma-Aldrich) was added to the tube and mixed. The mixture was incubated for 30 min at 37°C. Then 10 µl proteinase K (20 mg/ml), 100 µl 10% SDS, and 20 µl RNase A (20 mg/ml) were added, vortexed thoroughly, and incubated for 45 min at 55°C. Microbial cells were lysed by mechanical disruption using a bead beater (FastPrep instrument, Qbiogene) set at 6.0 m/s for 40 sec. DNA was purified from the lysate using the ZR Fecal DNA extraction kit (ZYMO Research) and according to the manufacturer's protocol omitting the lysis steps (steps 1–3). The kit includes a column (Zymo-Spin IV-HRC spin filter) specifically designed to remove PCR inhibitors from the DNA samples. The DNA was eluted into 100 µl of TE buffer, pH 8.0. This procedure provided between 2.5 and 5 µg of high quality whole genomic DNA from vaginal swabs measured using the Quant-iT PicoGreen dsDNA assay kit from Molecular Probes (Invitrogen).

#### (ii) Pyrosequencing of barcode 16S rRNA genes for community composition analysis

Universal primers 27F and 338R were used for PCR amplification of the V1-V2 hypervariable regions of 16S rRNA genes [21]. The 338R primer included a unique sequence tag to barcode each sample. The primers used were 27F-5′-GCCTTGCCAGCCCGCTCAGTC**AGAGTTTGATCCTGGCTCAG**-3′ and 338R-5′-GCCTCCCTCGCGCCATCAGNNNNNNNNCAT**GCTGCCTCCCGTAGGAGT**-3′), where the underlined sequences are the 454 Life Sciences FLX sequencing primers B and A in 27F and 338R, respectively, and the bold letters denotes the universal 16S rRNA primers 27F and 338R. Pyrosequencing of barcoded 16S rRNA gene amplicons was performed using the same method described by Ravel *et* *al*.[22]. The QIIME software package [23] was used for quality control of the sequence reads using the split-library.pl script and the following criteria: 1) no ambiguity base 2) minimum and maximum length of 220 bp and 400 bp; 3) an average of q25 over a sliding window of 50 bp. If the read quality dropped below q25 it was trimmed at the first base pair of the window and then reassessed for length criteria; 5) a perfect match to a barcode sequence; and 6) presence of the 338R 16S primer sequence used for amplification. Sequences were binned based on sample-specific barcode sequences and trimmed by removal of the barcode and primer sequences (forward, if present, and reverse). High quality sequence reads were first de-replicated using 99% similarity using the UCLUST software package [24] and detection of potential chimeric sequences was performed using the UCHIME component of UCLUST [25]. Chimeric sequences were removed prior to taxonomic assignments.

**Figure 2:**
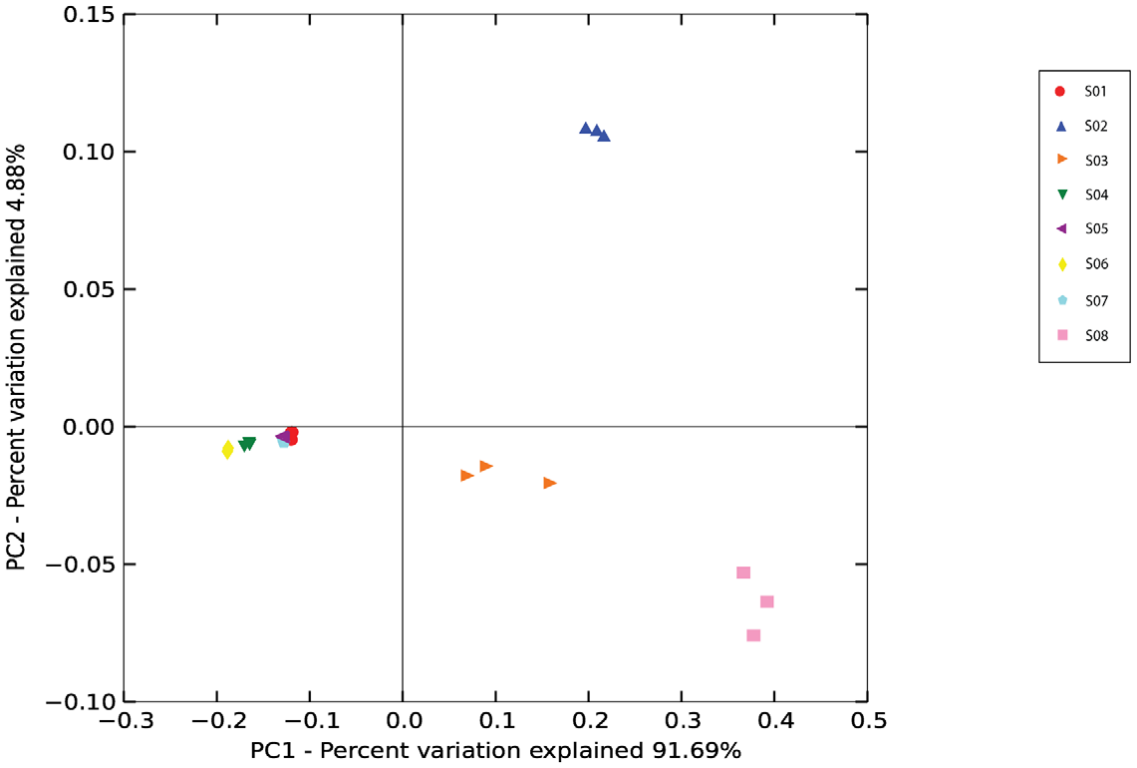
Distribution of 16S rRNA sequence data in UniFrac principal coordinates analysis (PCoA). The symbols represent the 23 samples from 8 subjects summarized in Table 1 and 2. Symbols are colored by subject (shown in legend). The scatterplot is of principal coordinate 1 (PC1) plotted against principal coordinate 2 (PC2). The percentage of the variation in the samples described by the plotted principal coordinates in indicated on the axes.

Genus level taxonomic assignments were performed by using the RDP Classifier [26], and further species level assignments for *Lactobacillus* sp. were done using 127 HMM *Lactobacillus* species models followed by clustering analysis using the software speciateIT (speciateIT.sourceforge.net).

**Figure 3:**
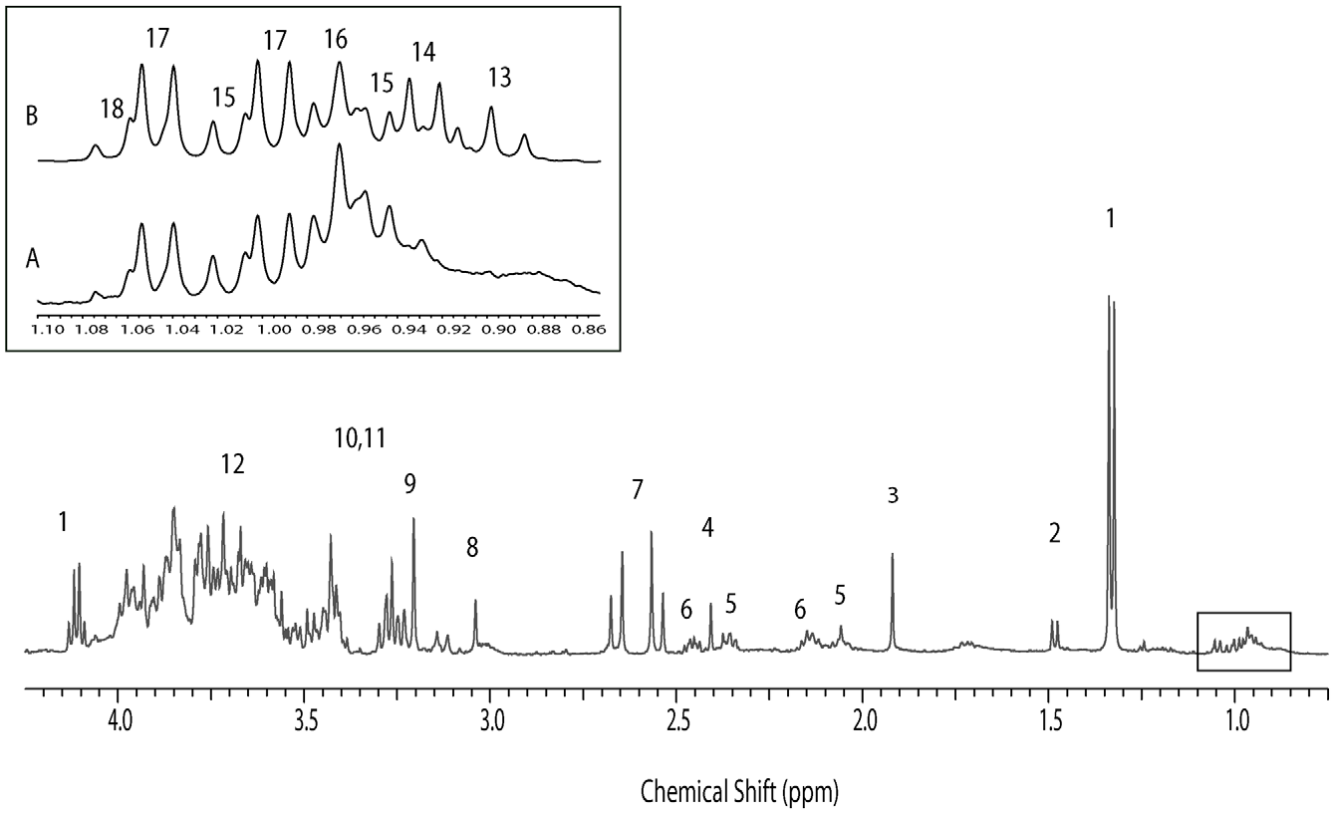
^1^H NMR spectrum for subject #S08 at condition 1. Enlarged spectral region of δ = 0.85–1.10 ppm from S08 in inset A and S03 in inset B. The major metabolites are labeled: 1) lactate; 2) alanine; 3) acetate; 4) succinate; 5) glutamate; 6) glutamine; 7) citrate; 8) creatine; 9) choline; 10) glucose; 11) maltose; residual lubricant ingredient 12) PEG; 13) *n*-butyrate; 14) iso-valerate; 15) Isoleucine; 16) leucine; 17) valine and 18) propionate.

**Figure 4:**
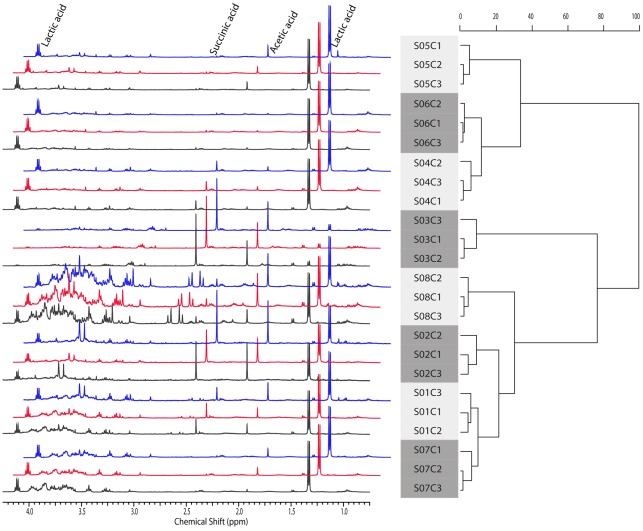
One-dimensional ^1^H NMR spectra of 24 aqueous vaginal samples from 8 participants (S01-S08) stored under three different conditions (C1 in black, C2 in red, and C3 in blue). The Ward hierarchical clustering of samples was based on the normalized spectral integrals representing the relative proton abundance of the metabolites.

**Table 3:** The relative abundance of major metabolites in vaginal samples.

sample ID	Condition	Community Type	Percentage of metabolites (%)		
			Lactic acid CH_3_ *δ* = 1.33 ppm	Acetic acid CH_3_ *δ* = 1.92 ppm	Succinic acid 2CH_2_ *δ* = 2.41 ppm
S01	1	II	9.6	1.6	2.8
	2	NA	12.0	3.4	2.7
	3	II	11.7	2.4	3.0
S02	1	IV	11.6	5.0	6.2
	2	IV	16.8	5.2	8.1
	3	IV	10.8	5.6	8.2
S03	1	IV	3.0	7.5	16.7
	2	IV	3.1	7.8	17.3
	3	IV	3.0	6.0	11.9
S04	1	I	23.0	2.2	4.3
	2	I	19.4	2.4	3.9
	3	I	23.8	2.4	4.4
S05	1	III	17.6	2.1	1.9
	2	III	16.7	1.8	1.9
	3	III	16.3	2.6	2.0
S06	1	I	25.7	1.6	2.3
	2	I	26.9	1.5	2.3
	3	I	24.7	1.5	2.1
S07	1	III	15.1	1.0	1.6
	2	III	15.4	1.7	1.9
	3	III	14.4	1.8	1.9
S08	1	IV	6.3	1.3	1.6
	2	IV	6.1	2.3	1.7
	3	IV	6.2	2.2	1.5

#### (iii) Statistical comparative analysis

For each sample, vectors of phylotype proportions were clustered into community state types as previously reported by Ravel *et* *al.* [22]. We sought to test the hypothesis that there were no significant differences between a woman's samples stored under the three cold chain conditions. The vaginal bacterial compositions obtained from two different storage conditions within a women were compared by computing the Jensen-Shannon divergence between the two vectors of phylotype proportions representing the community compositions [27], which is a measure of the distance or dissimilarity between these two communities. In order to estimate the extent to which community states of samples stored at two different storage conditions differ from each other, we compared the distances between these community states to the distances between community states of samples stored at the same storage condition. The Kolmogorov-Smirnov test [28] was used to show that at the 0.05 significance level, the null hypothesis that these two samples of distances came from the same distribution cannot be rejected. The null distribution of distances between community states of samples stored at the same storage condition was estimated utilizing data from prior work in which sixteen vaginal sample triplets were collected, each triplet was collected from the same women by a physician [19]. In order to demonstrate that the results were not sensitive to the choice of a dissimilarity measure between community states, the Kolmogorov-Smirnov tests were applied to the following measures of dissimilarity: relative entropy, Euclidean distance, Euclidean distance applied to log transformed relative abundances, Bray-Curtis metric.

Previous studies have suggested that comparison of communities should be made using equal number of sequence reads in order to minimize the sequencing artifact as the number of spurious phylotypes increases with sequencing effort [29]. We have randomly picked 4000 sequencing reads from each sample using a pseudo-random generator [30], [31] for a secondary comparison of community composition and structure among samples. 16S rRNA amplicon sequences were analyzed using the QIIME (v 1.4.0) suite of software tools [23]. Similar sequences with less than 1% dissimilarity were clustered together and detected chimeras were removed using the usearch method [24]. The processed sequences were then aligned using Python Nearest Alignment Space Termination (PyNAST) [32], and gaps and parsimonious phylogenetically uninformative characters in the alignment were removed. The taxonomic ranks were assigned to each sequence using RDP classifier v.2.2 [26] with 0.8 confidence values as the cutoff to a pre-built greengenes database of assigned sequences (February 4, 2011 version) [33]. The *de novo* phylogeny was built based on filtered alignment using RAxML method [34], and the phylogeny-based weighted UniFrac distance metrics [35] were calculated to assess the difference in overall microbial community composition. To provide visualization of the sample distribution patterns, a principal coordinates analysis (PCoA) was then used to transform the UniFrac distance matrices into principal coordinates.

### ^1^H NMR metabolome study

#### (i) ^1^H NMR sample preparation and data acquisition

Each sample consisted of one dry dacron Starplex swab head cut with ethanol-sterilized scissors and placed in a 1.5 ml centrifuge tube. Approximately 0.6 ml of deuterated water was added to the centrifuge tube as an extraction solvent. The samples were homogenized by vortex mixing for 1 min and stored on ice for 5 min. The solution was pipetted into a clean 1.5 ml tube and centrifuged (3 min, 13,000 rpm, 4°C) in a benchtop microcentrifuge to remove particulates. A sterile (unused) swab was also processed using the same procedure as extraction negative control.

^1^H NMR was used to establish the samples' metabolite profiles. A total of 450 µl of the resulting swab extract yielded a 500 µl sample containing 50 mM phosphate buffer at pH 7.0 and 30 µM sodium 3-(trimethylsilyl) propionate–2,2,3,3-d4 as internal chemical shift reference. The resulting solution was vortex mixed and then centrifuged at 13,000 rpm for 2 min and transferred to a 5 mm NMR tube. All ^1^H NMR experiments were carried out at 25°C on a Varian AS500 spectrometer operating at a proton NMR frequency of 499.75 MHz. One-dimensional spectra were recorded using a standard Carr-Purcell-Meiboom-Gill (CPMG) pulse sequence. An 80 ms CPMG pulse train was used to eliminate signals from large molecules, such as proteins from blood serum. Each spectrum consisted of 128 transients with a spectral width of 12 ppm and relaxation delay of 5.0 s. All free induction decays were Fourier transformed with an exponential function equivalent to a 0.3 Hz line-broadening factor and the spectra were zero filled to 32K points. The resulting spectra were manually phased and baseline corrected using ACDLABS (version 10.0, Advanced Chemistry Development, Inc.). For ^1^H NMR signal assignment purposes, two-dimensional (2-D) J-resolved spectroscopy [36] and total correlation spectroscopy (TOCSY) [37] NMR spectra were acquired for two selected samples. J-resolved spectra were collected using 128 scans per 32 increments with 5,000 Hz spectral width in F2 and 36 Hz in F1. The TOCSY spectra were recorded with a data matrix of 2048×128 with spectral width of 5,000 Hz in F2 and F1. Sixty-four scans were acquired and a mixing time of 80 ms was used. All 2-D NMR data were processed with the software package NMRPipe[38].

#### (ii) Metabolic composition and comparative analysis

The ^1^H-NMR spectra were reduced to 102 integrated regions of variable width corresponding to the region of δ  = 0.50–8.30 ppm using intelligent bucketing from ACDLAB. The region from δ  = 4.7–5.0 ppm was excluded from the analysis to avoid the phasing effects of the pre-saturation of the residual water signal. Normalization of the integrals to the total sum of the spectrum was carried out on the data prior to analysis (described below) to allow for differences in signal-to-noise. The normalized integrals were used as an indicator for the relative abundance of the metabolites in the aqueous vaginal solution.

#### (iii) Statistical analysis

For each pair of storage conditions (C1 vs C2, C2 vs C3, C1 vs C3), a Wilcoxon test [39] was performed on NMR integrals within different ranges of chemical shifts. In order to correct for multiple testing (over different chemical shift ranges), False Discovery Rates were computed for each integral over a range of chemical shifts.

## Results

The mean age of participants was 40.5 (range 25–63) and 62% self-reported African American ethnicity (**Table 1**). Seventy-five percent reported one sex partner in the prior 60 days and 75% reported feminine hygiene product use in the prior 60 days.

Of the 23 samples analyzed for microbiome bacterial composition, the twenty most abundant bacterial species are shown in **Figure 1** and **Table 2**. The communities were most often dominated by one or more species of *Lactobacillus*. Communities in community state type (CST) I were dominated by *L. crispatus*, whereas groups II and III were dominated by *L. gasseri* and *L. iners*, respectively. In general, CST IV lacked significant numbers of lactobacilli and was characterized by higher proportions of anaerobic organisms including members of the bacterial genera *Prevotella, Atopobium, Megasphaera* and *Parvimonas*. Of the eight women, there were two women (25%) with high Nugent scores and both observations were categorized to CST IV. The Kolmogorov-Smirnov tests were used to compare the distances between community states for the following storage conditions within a woman: C1 vs C2, C1 vs C3 and C2 vs C3. *P* values were larger than 0.11, supporting the hypothesis that there were no statistically significant differences between dissimilarity measures for each pair of storage conditions. The results of other metrics (relative entropy, Euclidean distance, Euclidean distance applied to log transformed relative abundances, and Bray-Curtis metric) were the same as in the case of the Jensen-Shannon divergence measure.

A UniFrac-based PCoA plot also revealed a strong pattern of primary clustering of bacterial composition by participant (shown in Figure 2 ). The first principal component explained 91.69% of the variation, and the second and third principal components explained 4.88% and 2.1% of the variation, respectively. Samples collected from the same subject cluster together, and within-subject UniFrac distances were generally smaller than between-subject distances, suggesting the community composition of samples from the same subject were more similar to each other and consisted of bacterial lineages sharing a common evolutionary history. Furthermore, our results indicate CST IV samples (S02, S03, and S08) displayed greater distance than samples of CST I, II, and III. This finding corresponds to the heterogeneity in community composition of CST IV samples, which reflect a diverse array of facultative and strictly anaerobic microorganisms from various taxonomic groups of bacteria [40], [41], compared to CST I, II and III which are largely dominated by *Lactobacillus* species.

A heatmap of the relative abundance of metabolic content featured by NMR is also displayed in **Figure 1**. The 18 most distinguishable metabolites are labeled on one spectrum (**Figure 3**). The integrals of a series of NMR regions were used to quantify the content of metabolites contributing signals to these regions. The normalized values of these integrals represent the relative abundance of these metabolites. There were no statistically significant differences between the NMR data of any pair of the three storage conditions within subjects (*p*-value >0.13). Ward hierarchical clustering of the one-dimensional NMR spectra also display clustering of samples within women (**Figure 4**) and were based on the normalized spectral integrals representing the relative proton abundance of the metabolites.

Strong signals from lactic acid (*δ*  = 1.33 ppm and 4.11 ppm) were observed in the NMR data for most samples except subject #S03 and #S08. Subject #S03 had relatively high abundance of both *L. iners* and Gram-negative anaerobes including *Prevotella*, *Atopobium* and *Megasphaera*. #S08 was not dominated by *Lactobacillus* and had high proportions of strictly anaerobic bacteria, including *Prevotella*, *Atopobium* and *Sneathia*. The lactic acid abundance in subject #S04 was almost as high as #S06 despite the differences in abundance of *L. crispatus* and the combination of *L. crispatus*, *L. iners* and *L. jensenii*. Subject #S01, dominated by *L. gasseri*, had a relatively low lactic acid level. (**Table 3**) Overall, women observed to have relatively low *Lactobacillus* abundance (CST IV, #S02, #S03, #S08) were among the women with the lowest lactic acid levels (calculated using NMR signals of the methyl group in lactate). Eighty nine percent of samples classified to CST IV had 3–12% lactic acid levels, as compared to women dominated by *L. crispatus* who had higher concentrations of lactic acid (18–27%).

## Discussion

We compared three cold chain protocols and their effect on the analysis of both the microbiome and metabolome of mid-vaginal samples. No significant differences were observed, indicating that storage at ultra-low temperature (−80°C), or storage for one week at −20°C prior to storage at −80°C for 4 weeks, did not significantly affect either the microbial or the metabolic composition of the vaginal samples when compared to samples from the same woman that were processed within 3 hours of collection without freezing. These findings validate epidemiologic studies of the vaginal microbiome in which swab samples are collected at offsite field clinics or self-collected at home.

Our study is consistent with several studies on other microbial systems that have shown similarities across storage conditions. Dolfing *et* *al*. [1] and Klammer *et* *al*. [2] reported high similarity of the overall structure of soil bacterial communities using DNA fingerprinting methods regardless of storage conditions. Nechvatal *et* *al*. found that fecal DNA was well preserved after being stored at room temperature in several preservatives for at least 5 days versus samples frozen in liquid nitrogen [3]. Roesch *et* *al*. found little change in stool bacterial community diversity after 72 hours at room temperature before freezing [4]. Lauber *et* *al*. evaluated the effect of storage conditions the bacterial composition of soil, human feces and skin using pyrosequencing of barcoded 16S rRNA gene sequence analysis. They found no significant differences in the phylogenetic structure and diversity of communities in individual samples stored at 20, 4, −4 or −20°C for 3 or 14 days [5]. In contrast to the above studies showing consistency across a variety of storage conditions and sample types, Tzeneva *et* *al*. [6] and Ott *et* *al*. [7] reported significant effect of storage conditions (room temperature and 4°C) on the composition and diversity of microbial communities in soil and human fecal samples, respectively.

We found that the metabolite composition of vaginal samples was unaffected by varying cold chain protocols. While it has been recommended that tissue samples be frozen in liquid nitrogen prior to storage at ultra-low temperature in order to maintain their chemical integrity [8], biofluids, such as urine and blood serum, appear not to require instant deep-freezing. Several studies [9], [10], [11] have shown that urine and serum samples were biochemically stable after being stored at 4°C for 24 hours, and that short term deep freezing at −80°C did not affect their metabolic profiles. Saude *et* *al*. [12] evaluated the effect of storage conditions (22 °C, 4°C and −80 °C for up to 4 weeks) on urine metabolites and found significant changes for a number of metabolites when samples were stored at 22°C, while the changes were smaller at 4°C and storage at −80°C provided a metabolite profile that best reflected the original samples. A gas-liquid chromatography study of the short-chain fatty acids in feces, a bacteria-rich biological sample, showed no significant difference between samples analyzed immediately after collection and those stored at −20°C for seven weeks [13]. In contrast, after freezing fecal samples, a NMR-based metabolomic study observed visible changes in short chain fatty acids [14]. Differences in sampling methods certainly explain these different results. In human biological samples, which are rich in microbes, unlike blood and most urine samples, one could imagine that sample collection, hence environmental change (for example, anaerobic to aerobic conditions) would trigger a shift in the metabolism of the indigenous microbiota that could alter the chemical and metabolic composition of the samples.

To our knowledge, this is also the first report of the combined analysis of the vaginal microbiota and its small molecule metabolites. While we were able to evaluate the effect of storage conditions on the metabolic make up of the samples, we had the opportunity to study the abundance of lactic acid and that of lactic-acid producing *Lactobacillus* sp. Lactic acid is thought to be the primary vaginal acidifier [42], [43]. Lactic acid has also been shown to be more effective than pH alone at inhibiting bacterial growth and preventing HIV [44], [45]. We observed the presence of lactic acid, acetic acid and some other small molecule metabolites (Figure 3 and Table 3). Lactic acid was most abundant in the samples of subject#S06, who was classified by microbiome analysis as being dominated by *L. crispatus*. Subject #S03 had the lowest content of lactic acid even though the participant had a relatively high level of *L. iners* (near 50%). We hypothesize that *L. crispatus* may be able to produce more lactic acid than other *Lactobacillus* species. However, due to the small number of samples analyzed, caution should be taken in interpreting these results. Because of the extent of genomic diversity within microbial species, it is possible that certain strains of *L. iners* are good acidifiers, and conversely, some strains of *L. crispatus* could be poor acidifiers. Of note in this study is the finding that samples from the group IV community state type contained lactic acid in varying abundance. CST IV samples have lower abundance of *Lactobacillus* sp., yet also had higher numbers of other lactic acid bacteria such as *Atopobium* spp. and *Megasphaera* spp. This suggests that the production of lactic acid may be present in a low-*Lactobacillus* state although levels of lactic acid were not as high as in the *Lactobacillus*-dominated communities (3–12% vs 18–27%).

Subject#S03 had the highest levels of acetic acid and succinic acid (**Table 3**), which have previously been detected in high abundance in the vaginal fluid of women with BV [46], [47]. Samples from#S03 also contained significantly higher levels of *n*-butyric acid and propionic acid (Figure 3) which certain anaerobic bacteria such as *Prevotella* and *Mobiluncus* spp. have the ability to produce [47]. It has been shown that the concentrations of short chain fatty acids, such as acetate, butyrate and propionate are significantly higher in BV-positive women when compared to women without BV [48], [49]. The high Nugent score of sample#S03 (**Figure 1**) indicated high BV risk, which supports these NMR findings.

In summary, varying cold chain protocols did not affect microbiome and metabolomic profiles of vaginal specimens, a finding that greatly benefits reproductive health studies, which utilize field sites or self-collection of specimens in the home setting. Preliminary data using ^1^H NMR spectroscopy of vaginal secretions confirms that lactobacilli are robust, with varying degree, in their production of lactic acid and that vaginal bacterial communities lacking significant numbers of *Lactobacillus* sp. also produce a modicum of lactic acid. A critical area of research remains the functional differences between vaginal community state types and their association with reproductive health outcomes including STI acquisition, and development of pelvic inflammatory disease and adverse obstetric outcomes. Future research should engage the factors that lead to development and maintenance of specific vaginal microorganisms and their role in mucosal protection from pathogens [50]. This research validates the use of samples collected in a home setting for microbiome and metabolome analysis.
